# Treatment for Tape-Worm, from Squibb’s Ephemeris

**Published:** 1883-01-20

**Authors:** 


### TREATMENT FOR TAPEWORM.

*From Squibb's Ephemeris.*

The writer has for many years past received occasional letters of inquiry as to what is the best drug for the expulsion of the tapeworm, and the inquiry is generally accompanied by the statement that a case is under treatment which had resisted all the ordinary parasiticides, such as pumpkin-seed, male fern, kooso, bark of pomegranate root, turpentine, etc. As any one of the drugs is sufficient, under any ordinary good management, to expel the parasite, and as the inquirers had generally succeeded in most of their cases with some one or other of these medicines, it has generally been concluded that it was not a question of the choice of a drug in the obstinate cases, but rather one of the location of the attachment of the head of the worm in the intestinal canal.

When the writer served as demonstrator of anatomy many years ago, he observed that there was great variation in the location of the head. Sometimes it would be found attached up near the duodenum, and at other times down near the ilio-cæcal valve, and that the attachment was not unfrequently in a little pouch, or under a fold of mucous membrane; and that the head was always imbedded in a nidus of firm jelly-like substance, like inspissated mucus. This led to the conclusion that such cases would be very differently affected by treatment, and that a method quite efficient for some cases would be likely to fail in others from the difference of location; and further, that the obstinate cases were those where the attachment was so low down in the canal, and so protected that it was difficult to get the parasiticide in contact with the head so as to poison it, and cause it to let go its hold. A few years later when in the eastern part of the Mediterranean, where uncooked sausages are largely eaten, the writer and others became affected with tapeworm, and he had good opportunities for observation, and was confirmed in the belief that the location of the head had much to do with the resistance of all obstinate cases, and that when the treatment was carefully directed by this consideration it was almost always successful, and that one parasiticide was about as good as another when well managed. Further experience at that time seemed to show that pumpkin-seed and oleoresin of male fern were the best agents to use, and that there was but little choice between them. A plan of treatment was adopted which has been since given to so many physicians and patients with such general success that it may be worth while to publish it.

After a light dinner, near the middle of the day, the patient should take no food, but may drink freely of water. At bed-time a saline aperient should be taken in effective dose, and there is nothing better than one or two Seidlitz powders. This aperient should be a saline, because these cause a copious effusion of serous liquid from the whole mucous membrane of the canal, and this effusion taking place from the surface where the head of the worm lies protected by the dense mucus, detaches the mucus and washes it away, leaving the head bare for contact with the parasiticide, when otherwise it would pass over it without direct contact, and, therefore, without effect.

Whether this aperient at bed-time operates or not, it should be repeated on the following morning, the patient still abstaining from food. After the second saline has operated freely, or say at about 10 o'clock the medicine should be given.

Four ounces of pumpkin-seeds are well beaten in a mortar, half an ounce at a time, a few drops of water being added from time to time until they are made into a paste. The shells need not be rejected, as they are rather useful than hurtful. Water is then gradually added to the paste with trituration, until a tolerably uniform emulsion is made measuring about a pint. This may be flavored if desired and iced, and is to be given in three doses at intervals of about two hours, beginning at about ten o'clock. During this time the patient should lie quietly in bed and avoid all causes of nausea or vomiting, and should correct these if they occur by a little ice taken into the mouth and stomach. The stomach in need of food will often digest the first dose, but a tendency to nausea will prevent the digestion of the others, and the third is often difficult to take without vomiting, By careful management and quiet the inverted peristaltic action may be generally avoided. But when it occurs early and is persistent the treatment is likely to fail, because the inverted action of the bowel prevents the emulsion from getting far enough down to come in contact with the head of the worm. Commonly, however, the peristaltic action will not be reversed, and about the time of the third dose or a little later there will be an alvine evacuation. Bnt if not within an hour after the last dose, a half fluid ounce of castor oil should be given in a little ale or porter. The evacuations should be received in a vessel partly filled with water so that the worm can be easily examined from end to end of each portion without breaking, and when the part is reached where the links grow smaller great care should be taken to find the head, for unless this be found the success of the treatment is by no means assured. And if the head be not found, detached links may be expected in the stools within three or four months, and the treatment will need to be repeated with larger preliminary fasting and greater care.

In a second trial, or when persistent vomiting has interfered with the first to invalidate it, the oleoresin of male fern may be substituted for the pumpkin seed. This is more easily taken than the large doses of emulsion, and is not so easily digested by the stomach, nor so liable to produce nausea, and from being an oleoresin, and therefore less soluble in the liquids it meets with, it is more likely to reach the head of the worm in a condition sufficiently concentrated to be a poison to the head, but it is probably a less active poison to the head than the pumpkin-seed, and therefore less active.

The oleoresin may be given in emulsion made with sugar and gum arabic, or with glycogen, but is perhaps better given in capsules, containing about ten grains each. Two of these should be taken every quarter of an hour until twelve capsules have been taken, unless nausea occurs of sufficient severity to endanger their rejection. Under such circumstances eight or ten capsules may be used as being all that can be safely given.

The oleoresin has often, especially in cold weather and when of good quality, a thick granular sediment. This should be carefully stirred in before weighing, as it is a very important part of the drug.

Of course the same careful preparation of the patient is needed with this as with the pumpkin-seed, and neither of them should be expected to succeed in obstinate cases without the careful preliminary treatment.

[We have succeeded in several cases with a single dose of pulverized kooso given on an empty stomach at morning, the patient having abstained from supper. We regard the kooso the best and surest of all the remedies used for the expulsion of tapeworm. The drug, as found in the shops, is often without strength from long keeping. The pulverized article kept in tight bottles is reliable.—ED. REC.]

